# Progress in the treatment of knee osteoarthritis with high tibial osteotomy: a systematic review

**DOI:** 10.1186/s13643-021-01601-z

**Published:** 2021-02-14

**Authors:** Mingliang He, Xihong Zhong, Zhong Li, Kun Shen, Wen Zeng

**Affiliations:** 1 Department of Orthopaedic Surgery, The Second Affiliated Hospital of Chengdu Medical College, China National Nuclear Corporation 416 Hospital, Chengdu, 610000 Sichuan China; 2 Affiliated Hospital of Southwest Medical University, Luzhou, China

**Keywords:** Knee osteoarthritis, HTO, OWHTO, CWHTO

## Abstract

**Background:**

High tibial osteotomy (HTO) has been used for over 60 years in clinical practice and mainly comprises two major techniques: closed wedge high tibial osteotomy (CWHTO) and open wedge high tibial osteotomy (OWHTO). However, these have been gradually replaced by total knee arthroplasty (TKA), due to inconsistent clinical results and many complications. With the concept of knee-protection and ladder treatment of osteoarthritis, as an effective minimally invasive treatment for knee osteoarthritis, HTO has once again received attention.

**Methods:**

A systematic literature search was conducted in PubMed, Embase, ClinicalKey, CNKI, and the China Wanfang database. The search terms relating to osteoarthritis and high tibial osteotomy were used. Studies were considered eligible if the participants were adults with knee osteoarthritis (KOA) who had undergone HTO. A total of two reviewers participated in the selection of the studies. Reviewer 1 was assigned to screen titles and abstracts, and reviewer 2 to screen full-text data. Data extraction was completed by reviewer 2, and 30% were checked by the research team. Potential conflicts were resolved through discussion. The methodological quality was assessed using a risk of bias, based on the Cochrane handbook and Newcastle-Ottawa assessment scale. The outcome indicators are (1) posterior slope of tibial plateau, (2) the height of the patella, (3) fracture in the osteotomy plane, (4) survival rate, (5) special surgery knee score (HSS), and (6) the recurrence of varus deformity of the included studies were evaluated according to the guidelines of the Grading of Recommendations, Assessment, Development, and Evaluation (GRADE) working group (Atkins et al., BMJ 328:1490, 2004).

**Results:**

Among the 18 articles included, 10 were prospective cohort studies, five were randomized controlled trial (RCT) studies, one was prospective comparative study (PCS), one was retrospective comparative study (RCS), and one was retrospective cohort. The earliest publication year was 1999, and the most recent was 2018. A total of 6555 eligible cases were included, comprised of 3351 OWHTO patients and 3204 CWHTO patients. Five RCT were assessed using risk of bias, based on the Cochrane handbook. Eleven cohort studies and two case-control studies were assessed using the Newcastle-Ottawa assessment scale. These six outcome indicators for a total of twenty-four evidence individuals were evaluated separately, among which the GRADE classification of 1, 2, and 6 was medium quality, and 3, 4, and 5 were low quality. Based on our systematic review, regardless of whether the chosen procedure was OWHTO or CWHTO, both HSS scores increased significantly as compared with the preoperative scores. Compared with CWHTO, the height of the patella and tibial posterior slope angle increased following OWHTO. Additionally, OWHTO has a better long-term survival rate and lower fracture rate, supporting OWHTO as the first treatment choice.

**Conclusions:**

For young patients with knee osteoarthritis (KOA), high tibial osteotomy (HTO) can be considered as a treatment option to replace total knee arthroplasty (TKA) to reduce the economic burden and promote the reasonable allocation of medical resources. This study shows that compared with CWHTO, OWHTO has certain advantages in long-term survival rate and lower fracture rate, but the level of evidence is lower. In the future, we will need larger sample sizes and longer follow-up randomized controlled trials to improve our research.

**Supplementary Information:**

The online version contains supplementary material available at 10.1186/s13643-021-01601-z.

## Background

Knee osteoarthritis (KOA) is a common orthopedic disease, most of which are accompanied by varus deformities of the knee joint [1]. Among people over 50 years old, KOA ranked second only to cardiovascular disease in terms of long-term disability [2]. KOA not only seriously affects the quality of life of patients, but also creates a heavy burden on society. There are currently approximately 355 million osteoarthritis patients worldwide. The number of osteoarthritis patients in China exceeds 100 million, of which the incidence of KOA is the highest, accounting for more than 30%. Among men older than 50 in the USA, the incidence of KOA is as high as 60–70%, which can cause a loss of 53% of the labor force; the annual economic loss caused by KOA amounts to US $ 5.46 billion [3].

At present, early KOA treatment is mainly symptomatic, in order to delay the progress of the disease, and the middle and late stages are primarily treated with surgery. The main surgical methods are TKA, unicompartmental knee arthroplasty (UKA), and HTO. According to statistics from Jacobs and Riddle et al. [4, 5], there is still 20% patient dissatisfaction following TKA. Those who are dissatisfied with the surgery tend to be younger patients with mild KOA symptoms. We know that in many KOA patients, the degenerative process is limited to the medial compartment, while the lateral and patellofemoral compartments are relatively intact [6]. For patients with single-compartment KOA, TKA is not worth the cost. Based on the theory of anterior medial osteoarthritis (AMOA), some experts have proposed UKA. Compared with TKA, UKA can retain more bone mass and does not require cutting the cruciate ligament in order to improve knee status. However, the indications for UKA are few. Osteoarthritis of the posteromedial compartment of the knee is usually accompanied by anterior cruciate ligament injury. If UKA is performed, the prosthesis will be unevenly stressed, and the knee joint will be unstable; the accelerated asymmetrical wear will increase the risk of revision. Also, UKA cannot correct deformities outside of the joint. HTO can correct the poor weight-bearing line, not only relieving pain and other symptoms; it is also a more conservative surgical procedure. HTO causes little interference with soft tissues and generally does not affect the stability and mobility of the knee joint. Santoso et al. [7] conducted a meta-analysis of 1013 HTO patients and 5438 UKA patients in 15 clinical centers. The results showed that there were no significant differences in walking speed, patellofemoral joint degeneration, revision rate, and hospital for special surgery knee score (HSS) between the two groups. However, HTO has a great advantage in terms of postoperative knee range of motion. The Smith team [8] compared the economic benefits of the three through modeling. They found that for medial knee osteoarthritis, patients under 60 years of age have the highest clinical benefit to economic burden ratio of HTO, and patients over 60 years are more suitable for UKA. It can be noted that HTO has its own advantages in treating young patients with medial knee osteoarthritis, providing a good prospect for clinical promotion.

Over the past 20 years, HTO has gradually become a research hotspot, with a large amount of literature and technological innovation. This paper summarizes the progress of research in recent years, from the surgical indications, the choice of surgical methods, the prevention of complications, the effect of HTO on the height of the patella, the long-term survival rate, and recurrence, providing a reference for the clinical application of HTO.

## Materials and methods

The protocol for the present review has been registered within the PROSPERO database (registration number: CRD4202020314). Our systematic review of the literature followed the PRISMA guidelines [9] (the PRISMA checklist is attached as a supplementary material) and established the exclusion criteria.

### Eligibility criteria

Studies were selected according to the following criteria: population, interventions, comparators, outcome(s) of interest, and study design (PICOS).

#### Type of studies

The types of studies are cohort, randomized controlled trials, controlled before-and-after studies, retrospective comparative studies, and prospective comparative studies.

#### Type of participants

Human participants are aged 18 years or older with osteoarthritis and having undergone high tibial osteotomy.

#### Type of interventions

The type of intervention is the use of HTO as a surgical method in treating KOA patients.

#### Type of comparison

KOA patients without surgical treatment are compared.

#### Type of outcome measures

The primary outcomes of interest were to compare the differences between OWHTO and CWHTO. We extracted and compared the data regarding the posterior slope of the tibial plateau, the height of the patella, fracture in the osteotomy plane, survival rate, HSS, and the recurrence of varus deformity.

The secondary outcomes only explored data collected during programme participation, primarily relating to surgical indications.

### Exclusion criteria

1. The full-text literature is not available or there is no detailed abstract;
2. Repeated publications or only periodic reports of a study.

### Search strategy

**Fig. 1:**
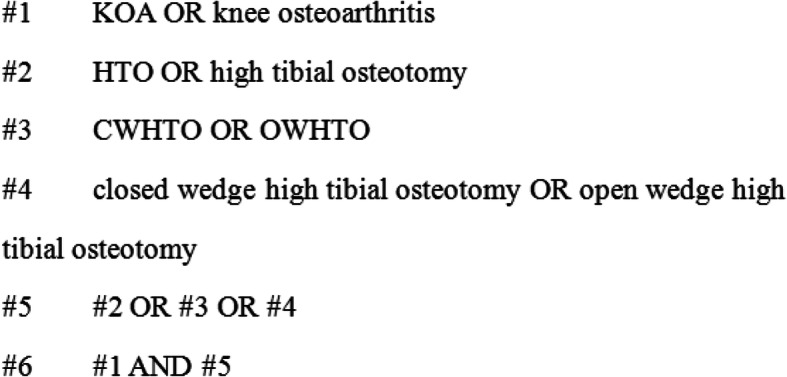
PubMed and Embase search strategy

**Fig. 2:**
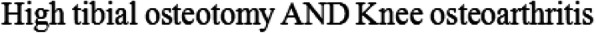
ClinicalKey search strategy

**Fig. 3:**
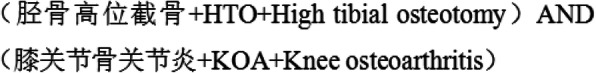
CNKI and the China Wanfang database search strategy

A librarian helped to develop the search strategy for the review. A systematic literature search was conducted in PubMed, Embase, ClinicalKey, CNKI, and the China Wanfang database. The search terms related to KOA, knee osteoarthritis, HTO, and high tibial osteotomy were used. Studies were sought by contacting experts in the field, references, and online website searching. The search time duration ran from the time of database construction to November 2018. The search strategy for each database were shown in Figs. 1, 2 and 3.

### Study selection

A total of two reviewers participated in the selection of the studies. Reviewer 1 screened titles and abstracts, while reviewer 2 screened full-text data. Data extraction was completed by reviewer 2, and 30% were checked by the research team. Potential conflicts were resolved through discussion.

### Quality assessment

Two independent reviewers assessed the accepted studies, assigning a level of evidence (from I to IV) using The American Academy of Orthopedic Surgeons classification system [10]. The methodological quality of the randomized controlled trials (RCT) was assessed using risk of bias (ROB), based on the Cochrane handbook, with the following seven standard criteria: (1) random sequence generation, (2) allocation concealment, (3) blinding of participants and personnel, (4) blinding of outcome assessment, (5) incomplete outcome data, (6) selective reporting, and (7) other bias (different follow-up period and rehabilitation methods). Each criteria were scored as “Yes (low ROB),” “No (high ROB),” or “Unclear.” The methodological quality of the cohort study or non-randomized case-control study was assessed using a Newcastle-Ottawa assessment scale. It consisted of three main domains (selection, comparability, and outcome), with four categories in the selection domain, one category in the comparability domain, and three categories in the outcome domain. A study was awarded a maximum of one star (*) for each item within the selection and outcome domains. A maximum of two stars was given for comparability. More stars meant a low ROB.

### Grading of the quality of the evidence

The outcome indicators of the included studies were evaluated according to the guidelines of the Grading of Recommendations, Assessment, Development, and Evaluation (GRADE) working group [11]. Using outcome indicators as evidence, individuals evaluated the outcome indicators of each systematic review based on five factors: limitation, inconsistency, indirectness, imprecision, and publication deviation. For the same outcome index, there may be different grade evidence levels due to different studies. We accept the lowest evidence level as the evidence level of this outcome index.

## Results

### Characteristics of included studies

**Fig. 4:**
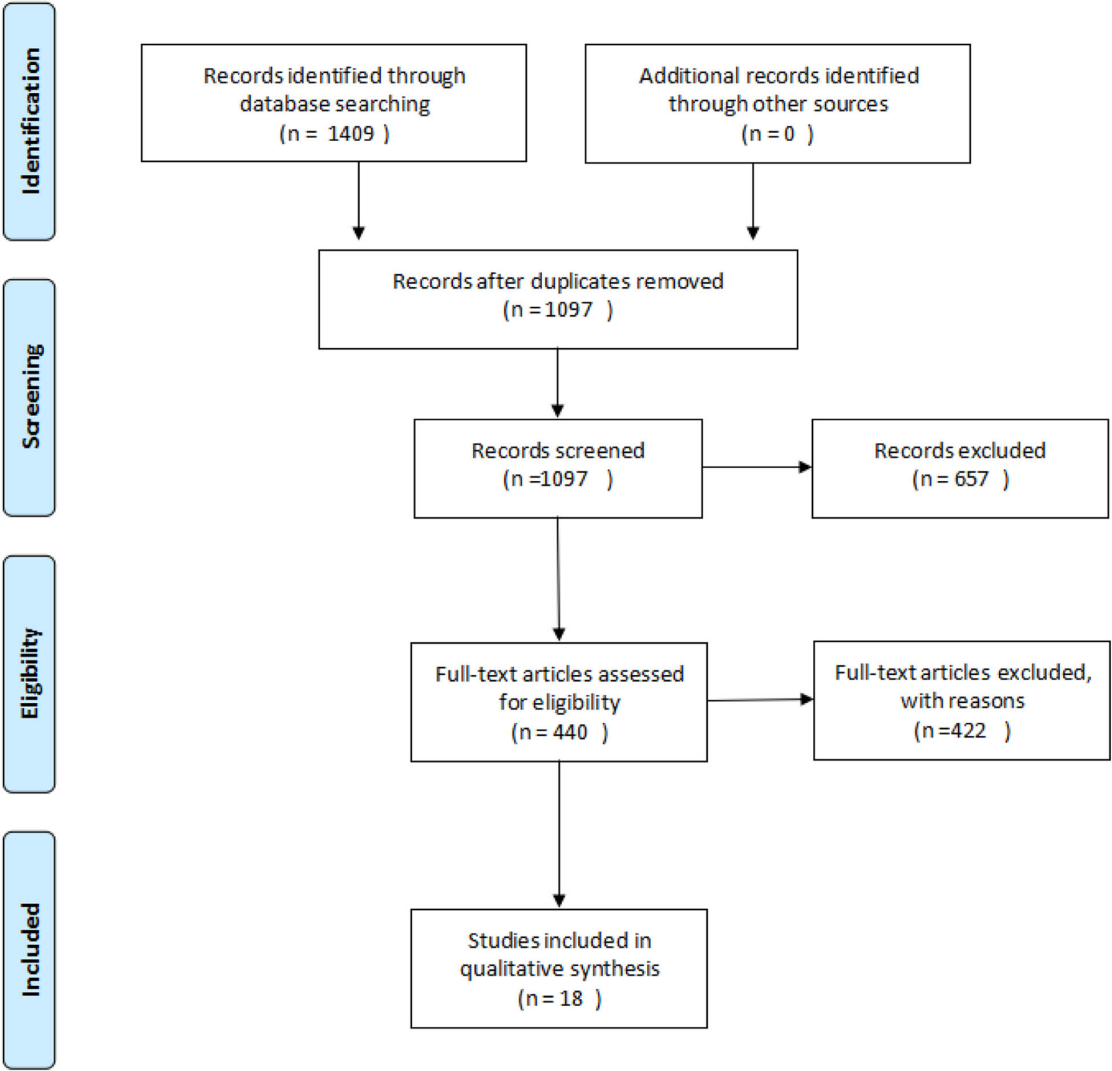
Literature screening flow chart

Among the 18 included articles [12–29], 10 were prospective cohort studies, five were randomized controlled trial (RCT) studies, one was a prospective comparative study (PCS), one was a retrospective comparative study (RCS), and one was a retrospective cohort. The earliest publication year was 1999; the most recent was 2018. Literature screening flow charts are shown in Fig. 4.

A total of 6555 eligible cases were included, comprised of 3351 OWHTO patients and 3204 CWHTO patients. The follow-up period varied between 8 and 97 months, with an average follow-up of 41.5 ± 5.6 months. The research characteristics of the included literature are shown in Table 1.

**Table 1:** Characteristics of the included studies

Author	Year	Level of evidence	Study type	Case	Age(years)	Control	Follow up time (months)	Included outcome indicators
Chiu [12]	1999	III	Prospective cohort	25 (M:F=20:5)	68.0±16.2			①
Kuwano [13]	2005	III	Prospective cohort	32 (M:F=3:29)	71.4			①
Kyung-wook [14]	2016	II	RCT	1260 OWHTO		603 CWHTO	39	①
Hohmanm [15]	2005	III	Prospective cohort	67 CWHTO (M:F=41:26)	M:36.6 F:39.4		8	①
Giffin [16]	2004	III	Prospective cohort	10 OWHTO	60–78			①
Noyes [17]	2005	IV	Prospective cohort	35 OWHTO	32.7			①
Raaij [18]	2008	II	RCT	43 OWHTO		44 CWHTO	24	③,⑥
Turkmen [19]	2017	III	PCS	6 OWHTO (single plane osteotomy)		6 OWHTO (biplane osteotomy)		③
Nakamura [20]	2017	III	RCS	111 OWHTO (M:F=22:89)	63.8±7.2			③
Chen [21]	2012	III	Prospective cohort	8 OWHTO	33			②
Ozkaya [22]	2008	III	Prospective cohort	16 OWHTO (M:F=4:12)	55		24	①,②
Lee [23]	2018	II	RCT	831 OWHTO		206 CWHTO	60	②
Altay [24]	2016	III	Prospective cohort	34 OWHTO	45.05±10.99		74.65±10.12	①,②,④
Terauchi [25]	2002	III	Prospective cohort	29 OWHTO (M:F=6:23)	66		96.2	⑤,⑥
El-Assal [26]	2010	II	Prospective cohort	58 OWHTO (M:F=21:37)	47.5		38	⑤
Park [27]	2017	IV	Retrospective cohort	30 OWHTO				②
Bae [28]	2016	II	RCT	69 OWHTO (M:F=2:67)	58.5±5.8	49 OWHTO (M:F=4:45)	94.2±2.6	④,⑤
Kim [29]	2017	IV	RCT	687 OWHTO		2296 CWHTO		④

① posterior slope of tibial plateau, ② the height of the patella, ③ fracture in the osteotomy plane, ④ survival rate, ⑤ HSS, ⑥ the recurrence of varus deformity

*RCT* Randomized controlled trial, *RCS* Retrospective comparative study, *PCS* Prospective comparative study

### Quality assessment of included studies

Quality assessment details are presented in Table 2. Five RCTs were assessed using risk of bias (ROB), based on the Cochrane handbook. Eleven cohort studies and two case-control studies were assessed using the Newcastle-Ottawa assessment scale.

**Table 2:** Quality assessment of the included studies

Risk of bias for RCTs										
Author	Year	Level of evidence	1	2	3		4	5	6	7
Kyung-wook	2016	II	U	U	N		N	Y	N	N
Raaij	2008	II	U	U	N		N	Y	N	N
Lee	2018	II	Y	U	N		N	Y	N	N
Bae	2016	II	Y	U	N		N	N	N	N
Kim	2017	IV	U	U	N		N	Y	Y	N
Newcastle-Ottawa assessment for cohort studies										
Author	Year	Level of evidence	Selection				Comparability	Outcome		
			1(**)	2(*)	3(**)	4(**)	1(**)	1(**)	2(*)	3(**)
Chiu	1999	III	*	*	*	*	**	*	*	*
Kuwano	2005	III	*	*	*	*	**	*	*	*
Hohmanm	2005	III	*	*	*	*	**	*	*	*
Giffin	2004	III	*	*	*	*	**	*	*	*
Noyes	2005	IV	*	*	*	*	**	*	*	*
Chen	2012	III	*	*	*	*	**	*	*	*
Ozkaya	2008	III	*	*	*	*	**	*	*	*
Altay	2016	III	*	*	*	*	**	*	*	*
Terauchi	2002	III	*	*	*	*	**	*	*	*
El-Assal	2010	II	*	*	*	*	**	*	*	*
Park	2017	IV	*	*	*	*	**	*	*	*
Newcastle-Ottawa assessment for case-control studies										
Author	Year	Level of evidence	Selection				Comparability	Outcome		
			1(**)	2(*)	3(**)	4(**)	1(**)	1(**)	2(*)	3(**)
Turkmen	2017	III	*	*			*	*	*	*
Nakamura	2017	III	*	*			*	*	*	*

A study was awarded a maximum of one star (*) for each item within the selection and outcome domains. A maximum of two stars (**) was givenfor comparability. More stars meant a low ROB

### Outcome indicators and GRADE classification

The six outcome indicators of 18 articles were evaluated according to GRADE. The GRADE evidence quality of each outcome is shown in Table 3. These six outcome indicators for a total of twenty-four evidence individuals were evaluated separately, among which the GRADE classification of 1, 2, and 6 were medium quality, and 3, 4, and 5 were low quality. Based on our systematic review, regardless of whether the chosen procedure was OWHTO or CWHTO, both HSS scores increased significantly as compared with the preoperative scores. Compared with CWHTO, the height of the patella and tibial posterior slope angle increased following OWHTO. Additionally, OWHTO has a better long-term survival rate and lower fracture rate, supporting OWHTO as the first treatment choice. The details are as follows.

**Table 3:** GRADE evidence quality for each outcome

N(study)	Design	Limitation	Inconsistency	Indirctness	Imprecision	Publication Bias	N	Quality
①(study)	Prospective cohort 7 RCT 1	Yes(-2)	NO	NO	NO	NO	1479	Low
②(study)	Prospective cohort 3 RCT 1 Retrospective cohort 1	NO	Yes(-1)	NO	NO	NO	919	Low
③(study)	RCT 1 PCS 1 RCS 1	NO	NO	NO	NO	NO	60	Medium
④(study)	Prospective cohort 1 RCT 2	NO	NO	NO	NO	NO	790	Medium
⑤(study)	Prospective cohort 2 RCT 1	NO	NO	NO	NO	NO	156	Medium
⑥(study)	Prospective cohort 1 RCT 1	NO	NO	NO	NO	NO	72	Low

① Posterior slope of tibial plateau, ② the height of the patella, ③ fracture in the osteotomy plane, ④ survival rate, ⑤ HSS, ⑥ the recurrence of varus deformity

*RCT* Randomized controlled trial, *RCS* Retrospective comparative study, *PCS* Prospective comparative study

#### Posterior slope of the tibial plateau

It was addressed in one level II study [14], six level III studies [12, 13, 15, 16, 22, 24], and one level IV study [17]. The level of evidence was low quality. The results of these eight studies all demonstrated that PSA are generally increased following OWHTO, and PSA is generally reduced after undergoing CWHTO. However, both the range of change was approximately 2°–5°, which had little effect on the biomechanics of the knee joint cruciate ligament.

#### The height of the patella

It was addressed in one level II study [23], three level III studies [21, 22, 24], and one level IV study [27]. The level of evidence was low quality. In the OWHTO group, 83.3% exhibited a significant decrease in patellar height, with a mean of 15% (*p* < 0.05). However, in the OWHTO group, the patellar height showed no change following surgery, with a Blackburne-Peel index (BPI) [mean − 0.02], and Caton-Deschamps index (CDI) [mean 0.02]). The changes in patellar height following high tibial osteotomy did not result in any adverse effect on short-term patient satisfaction.

#### Fracture in the osteotomy plane

It was addressed in one level II study [18] and two level III studies [19, 20]. The level of evidence was medium quality. Among three studies, the results of one level II study [18] and one level III study [20] showed that the incidence of fractures in OWHTO is significantly higher than that in CWHTO, namely, 82% and 35% (*p*<0.05), respectively. And one level II study [19] reported that the biomechanical properties of the biplane osteotomy were significantly better than the single plane osteotomy, effectively reducing the incidence of contralateral cortical fracture. The maximum load of a single plane osteotomy and biplane osteotomy were respectively, 84.0 ± 19.5 N and 146.9 ± 22.0 N, and the maximum spreading distance was 14.7 ± 2.9 mm and 19.1 ± 2.0 mm, respectively.

#### Survival rate

It was addressed in 1 level II study [28], 1 level III study [24], and 1 level IV study [29]. The level of evidence was medium quality. The results of these eight studies all demonstrated that whether the procedure was OWHTO or CWHTO, the 5- and 10-year survival rates had reached 84.0–97.1%; these results were satisfactory. One level IV study [29] reported that the pooled 5-year survival rates were 95.1% (95% CI: 93.1 to 97.1%) in open wedge HTO and 93.9% (95% CI: 93.1 to 94.6%) in closed wedge HTO. Although there was a 1.2% greater survival rate for open wedge HTO than for closed wedge HTO, this difference did not reach statistical significance (*p* = 0.419). Pooled 10-year survival rates were 91.6% (95% CI: 88.5 to 94.8%) in open wedge HTO and 85.4% (95% CI: 84.0 to 86.7%) in closed wedge HTO, indicating that open wedge HTO had a 6.2% greater survival rate 10 years after surgery than did closed wedge HTO (*p* = 0.002). No difference in 5-year survivorship was found between open- and closed-wedge HTO. However, the survival rate was higher in open-wedge HTOs than in closed wedge HTO 10 years post-surgery.

#### Special surgery knee score (HSS)

It was addressed in two level II studies [26, 28] and one level III study [25]. The level of evidence was medium quality. The results of these three studies all demonstrated that the function of the knee joint was noticeably improved, and the average postoperative score increased to 92 (range 71–100) from 62 (range, 51–73).

#### The recurrence of varus deformity

It was addressed in one level II study [18] and one level III study [25]. The level of evidence was low quality. The results of these studies showed that HTO patients with a lateral femoral deformity were 11% more likely to have varus deformity recurrence within 5–10 years following surgery.

## Discussion

High tibial osteotomy (HTO) surgery is currently considered suitable for the following patients: (1) young (generally less than 65 years old), with a large amount of activity and (2) symptomatic single-compartment osteoarthritis of the knee joint, with the presence of a bony internal valgus deformity (mainly extra-articular deformity). The deformity angle is less than 20°; (3) the knee joint activity is good and the flexion activity is ≥ 100.

At present, there is still much controversy regarding the age for the indication of HTO surgery. Trieb et al. [30] found that the risk of HTO surgery failure was positively correlated with age, and it increased by 7.6% for every year after age 65. Therefore, it is not recommended to perform HTO surgery for patients over 65 years of age. Billings et al. [31] and Flamme et al. [32] hold different views. They report that there are individualized differences in the improvement of knee joint pain and other symptoms after HTO, with no relevant relation to age factors. Therefore, they considered that surgical treatment is also feasible for elderly people, as long as they meet the HTO indications. At present, in clinical practice, consideration of suitability for HTO surgery is not based on age factors. However, considering the generally lower activity level in the elderly, the incidence of multi-compartment osteoarthritis and bone non-healing is higher than that of younger patients. We propose that for KOA patients, younger patients are more suitable for HTO.

Common peroneal injury is the most common nerve complication following CWHTO, with an incidence ranging from 0 to 20%. Although common peroneal nerve injury is not unique to CWHTO, there is clearly a higher risk of this injury than in OWHTO. The common peroneal nerve accompanies the fibular neck and then divides into deep and superficial branches. The high-risk area of the common peroneal nerve injury is within 4 cm below the fibular head, while the 6–8 cm below the fibular neck is a relatively safe area. We recommend performing the fibular osteotomy in this area [10].

**Fig. 5:**
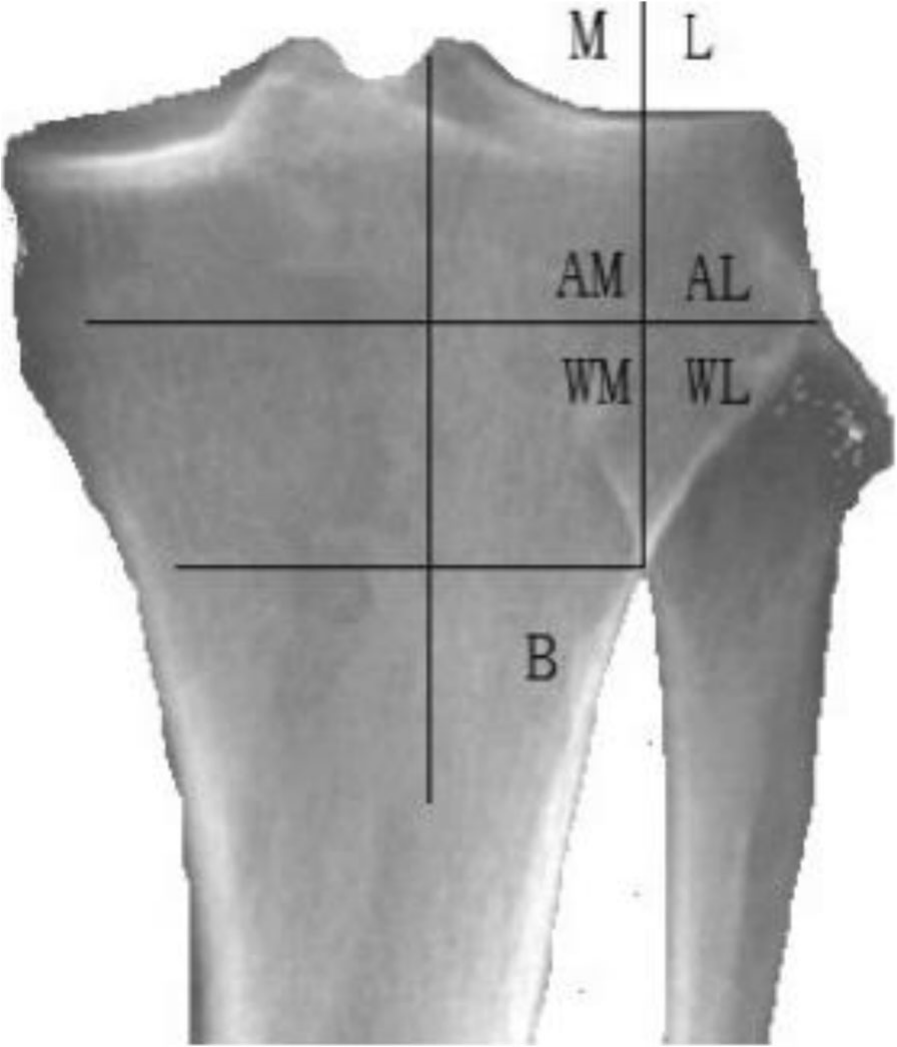
Proximal tibiofibular joint

Contralateral cortical fractures are another common surgical complication of HTO. Nakamura et al. [20] divided the proximal tibiofibular joint, where the hinge apex is divided into five areas: AL, AM, WL, WM, and B (Fig. 5) and then compared the fracture incidence in these five areas. The safest area was found to be the WL area, which can significantly reduce the incidence of contralateral cortical fracture. Even a team with significant experience, such as Nakamura’s team, still has a 20% fracture rate. Therefore, the consideration of the patient’s bone condition, the design of the hinge position, and the safety distance are necessary steps to prevent the contralateral cortical fracture.

HTO is primarily utilized to alter the weight bearing line deformity in the coronal plane; however, the posterior slope of the tibial plateau and the height of the patella in the sagittal plane are often ignored. At the same time, due to the morphological variation of the tibial plateau, there is no uniform conclusion regarding the exact value of the posterior slope angle (PSA) of the tibial plateau. The PSA of the medial tibial plateau in Europe and America is 11.9° (8°–16°) and of the lateral tibial plateau, 8.3° (4°–13°) [33]. The Chinese tibial plateau is more inclined, with an average of 14.8° (5°–25°) on the inside and 11.8° (4°–23°) on the outside [12]. Kuwano et al. [13] suggest that when the thickness of the osteotomy does not exceed 10 mm, and the lateral PS is used as a reference for osteotomy. They propose that this can reduce the amount of osteotomy. However, Chiu et al. [12] report that the medial PS is more closely related to the average of the tibial plateau PSA and that the osteotomy level should be predominantly medial. These data suggest that we should unify the measurement method and apply different adjustments to the tibial plateau correction degree according to different races when performing HTO preoperative planning.

At present, the importance of the posterior slope for the stability of the knee joint has received increasing attention. PSA reduction can impair the stability of the knee joint, resulting in over-extension of the knee and increasing the load on the posterior cruciate ligament. Conversely, as PSA increases, it also increases the anterior cruciate ligament load. It is clear that PSA is generally increased after OWHTO and PSA is generally reduced after CWHTO. Through the three-dimensional reconstruction of the proximal tibial, it can be seen that the proximal tibial is a triangular cylinder with a narrow front and a wide back, rather than a cylinder. The anatomical axis has a certain angle with the gravity axis. If the OWHTO is not strictly perpendicular to the anatomical axis, this can cause the front opening gap to be greater than the rear gap, increasing the PSA. If the CWHTO is not strictly perpendicular to the anatomical axis, it will lead to excessive osteotomy in front and a reduced PSA. Noyes et al. [17] found that the front osteotomy gap is approximately one half of the posterior osteotomy gap, which will maintain the PS unchanged. They also found that a difference of 1 mm between the front and back gaps would cause the PSA to change by 2. Therefore, in clinical practice, if we want to keep the PS unchanged, it is recommended to use a trapezoidal osteotomy, i.e., narrow at the front and wide at the back the CWHTO. However, in the OWHTO, the front opening gap and the rear-opening gap are maintained at a ratio of 1:2.

For cases of chronic posterior cruciate ligament injury, the posterior cruciate ligament (PCL) load can be reduced by increasing the PSA. The study by Savarese et al. [34] showed that the amount of change should be < 8°, and if the change value exceeds 8°, it will destroy the stability of the anterior cruciate ligament. At the same time, studies have shown an improvement in clinical results with a PSA change of > 5°. Therefore, for patients with knee osteoarthritis and a chronic PCL injury, we recommend setting the PSA correction at between 5° and 8°.

The recurrence of varus deformity was defined as an increase in the femoral tibial angle (FTA) of 3° or greater within 6 months after surgery [25]. The recurrence of a varus deformity will invalidate the surgery, increasing the risk of a secondary surgery to the patient, and seriously affecting the patient’s postoperative satisfaction.

If the patient’s deformity from the distal femur is not corrected, it is likely to cause recurrence of early postoperative varus deformity. This suggests that prior to the knee osteotomy, the source of the deformity should be identified. Examples include the distal femoral deformity in the coronal plane, the proximal tibial deformity in the coronal plane, intra-articular cartilage wear, and the bone defect in the distal femoral or the tibial plateau. For the deformity from the distal femoral, we can use the lateral distal femoral angle (LDFA normal value of 89°) to quantify. If the LDFA increases, it indicates there is a bony varus at the distal femur. For the deformity of the proximal tibial, we use the medial proximal tibial angle (MPTA normal value of 88°) to quantify. If the MPTA reduces, it indicates that there is a bony varus at the proximal tibial. If the MPTA and LDFA are both abnormal, we may need to perform HTO and a distal femoral osteotomy (DFO) combination surgery to correct the deformity. Deformities caused by cartilage wear or bone defects can be treated through cartilage repair and bone grafting. We believe that for patients with distal femoral varus deformity, HTO combined with distal femoral osteotomy (DFO) can achieve excellent clinical results after the early stage of surgery, but the long-term efficacy is still unknown and requires longer follow-up observation.

## Conclusion

For young patients with knee osteoarthritis (KOA), high tibial osteotomy (HTO) can be considered as a treatment option to replace total knee arthroplasty (TKA) to reduce the economic burden and promote the reasonable allocation of medical resources. This study shows that compared with CWHTO, OWHTO has certain advantages in long-term survival rate and lower fracture rate, but the level of evidence is lower. In the future, we will need larger sample sizes and longer follow-up randomized controlled trials to improve our research.

## Supplementary Information

**Additional file 1.** PRISMA 2009 Checklist.

## Data Availability

The data was all shown in the manuscript.

## Abbreviations

KOA: Knee osteoarthritis
TKA: Total knee arthroplasty
UKA: Unicompartmental knee arthroplasty
HTO: High tibial osteotomy
CWHTO: Closed wedge high tibial osteotomy
OWHTO: Open wedge high tibial osteotomy
ACL: Anterior cruciate ligament
PCL: Posterior cruciate ligament
PLC: Posterolateral complex
FTA: Femural tibial angle
LDFA: Lateral distal femural angle
MPTA: Medial proximal tibial angle
DFO: Distal femur osteotomy
PSA: Posterior slope angle
